# Within- and Between-Household Variation in Food Expenditures Among Low-Income Households Using a Novel Simple Annotated Receipt Method

**DOI:** 10.3389/fnut.2020.582999

**Published:** 2020-10-22

**Authors:** Sruthi Valluri, Simone A. French, Brian Elbel, J. Michael Oakes, Sarah A. Rydell, Lisa J. Harnack

**Affiliations:** ^1^Division of Epidemiology and Community Health, School of Public Health, University of Minnesota, Minneapolis, MN, United States; ^2^Medical Scientist Training Program, University of Minnesota Medical School, Minneapolis, MN, United States; ^3^School of Medicine and Wagner School of Public Service, New York University, New York, NY, United States

**Keywords:** nutrition methodologies, household food purchasing, food receipt method, within-household variation, between-household variation, epidemiology

## Abstract

**Background:** Household food purchasing behavior has gained interest as an intervention to improve nutrition and nutrition-associated outcomes. However, evaluating food expenditures is challenging in epidemiological studies. Assessment methods that are both valid and feasible for use among diverse, low-income populations are needed. We therefore developed a novel simple annotated receipt method to assess household food purchasing. First, we describe and evaluate the extent to which the method captures food purchasing information. We then evaluate within- and between-household variation in weekly food purchasing to determine sample sizes and the number of weeks of data needed to measure household food purchasing with adequate precision.

**Methods:** Four weeks of food purchase receipt data were collected from 260 low-income households in the Minneapolis-St. Paul metropolitan area. The proportion of receipt line items that could not be coded into one of 11 food categories (unidentified) was calculated, and a zero-inflated negative binomial regression was used to evaluate the association between unidentified receipt items and participant characteristics and store type. Within- and between-household coefficients of variation were calculated for total food expenditures and several food categories.

**Results:** A low proportion of receipt line items (1.6%) could not be coded into a food category and the incidence of unidentified items did not appreciably vary by participant characteristics. Weekly expenditures on foods high in added sugar had higher within- and between-household coefficients of variation than weekly fruit and vegetable expenditures. To estimate mean weekly food expenditures within 20% of the group's usual (“true”) expenditures, 72 households were required. Nine weeks of data were required to achieve an *r* = 0.90 between observed and usual weekly food expenditures.

**Conclusions:** The simple annotated receipt method may be a feasible tool for use in assessing food expenditures of low-income, diverse populations. Within- and between-household coefficients of variation suggest that the number of weeks of data or group sizes required to precisely estimate usual household expenditures is higher for foods high in added sugar compared to fruits and vegetables.

## Background

Food purchasing behavior has gained interest as an intervention target to improve nutrition and nutrition-associated health outcomes in the United States (1–4). Evidence that the nutritional quality of food purchases corresponds with dietary quality (5, 6) has prompted numerous interventions targeting food purchasing behavior (3, 4). Low-income populations have generated particular attention due to socioeconomic differences in diet quality (7, 8). However, valid and feasible methods for measuring food purchasing among low-income households are limited.

Existing methods to evaluate household food purchasing behavior—including home food inventories (9–12), bar code scanners (13, 14), point of sale data (15–17), food purchase records (18–21), and food receipts (22–25)—have unique strengths, but their weaknesses present noteworthy challenges for implementation, validity, and capturing the full range of purchase information (Table 1) (12). Furthermore, there are important differences in food purchasing by household socioeconomic status (7, 8). Assessments methods therefore need to be evaluated among low-income households to ensure that the detail and variation in household expenditures is fully captured (12).

**Table 1:** Description and summary of strengths and weaknesses of existing methods to assess household food purchasing.

**Method**	**Description**	**Strengths**	**Limitations**
Home food inventories (9–12)	Collected by study staff or participants. Catalogs foods available in the home at the time inventory is completed	• Low participant burden • Relatively easy to complete	• Multiple administrations of the inventory required for accurate assessment of usual household food available • Foods purchased and consumed outside home are not ascertained • Captures types of food (e.g., soft drinks) but not quantity (e.g., fluid ounces)
Bar code scanners (12–14)	Participants scan bar codes for all foods purchased. Researchers provide codes to participants for unpackaged items.	• Does not require participant literacy • Can provide rich data on types and quantities of packaged foods	• Scanners can be expensive, susceptible to hardware malfunctioning, and rely on external database of codes to match bar codes to food items. • May not capture foods that typically lack bar codes such as bulk items, fresh produce and meats in grocery stores, and food purchased at restaurants.
Point of sale data (12, 15–17)	Uses data available from food retailers on customer food purchasing	• Minimal participant burden • Can provide rich data on types and quantities of foods	• Linking data from vendors with individual shoppers can be challenging due to proprietary nature of data, privacy concerns, and technological issues • Unable to capture comprehensive assessment of household food purchases since it is generally limited to one retailer
Food purchase record (12, 17–21)	Participants keep a written record of all foods purchased, including description of each item and quantity.	• Offers detailed and comprehensive information about types and quantities of food purchased over time	• Requires participant literacy • High participant burden
Food receipt collection (12, 22, 23)	Participants collect and mail all receipts for food purchases. Receipt purchases are coded by study staff.	• Offers details about expenditures over time • Low participant burden	• May not be able to code purchases with insufficient detail on receipt, including specific types of food (e.g., “produce” vs. “tomatoes”) and quantities (e.g., fluid ounces)
Annotated food receipt (12, 24, 25)	Participants collect receipts for food purchases and transcribe receipt information onto a form to provide details not available on receipts	• Offers detailed information about all food and beverage purchases	• Requires participant literacy • High participant burden

Food receipt methods are appealing because they can be used to assess food expenditures from a variety of retailers and for all types of foods (12, 22–25). In the annotated food receipt method, participants collect receipts and transcribe information onto forms to clarify missing details and unclear abbreviations (e.g., items described as “dairy” rather than “skim milk,” or “Pillsbury white cake mix” listed as “pills white”) (24, 25). However, participant burden is high and literacy is required. In contrast, participants submit receipts without transcription or annotation in the food receipt collection method (22). Although this method substantially lowers participant burden, many details that receipts generally lack may not be captured.

To capitalize on the detailed information possible using the annotated food receipt method while reducing participant burden, a simple version of the annotated food receipt method (the “simple annotated receipt method”) was developed for use in a prospective trial (26). Participants are not required to transcribe all purchase information using this newly developed method; instead, they annotate items with vague or unclear descriptions directly on the receipts.

This study has two primary aims. First, we describe and evaluate the simple annotated receipt method using data from the aforementioned trial. We illustrate the food purchasing information that may be captured using this method. We also evaluate the extent to which receipt items could not be identified due to inadequate annotation and whether this varies by participant characteristic and store type. To date, this is the first study to describe and evaluate this method.

Second, we evaluate sources of variation in household food purchasing to help guide study designs using this method. Household food purchasing behavior is often evaluated for one of three research objectives: (1) to compare mean household expenditures between different groups (e.g., control vs. intervention groups), (2) to rank households by expenditures (e.g., into quartiles), (3) or to assess an individual household's expenditures (e.g., change in expenditures before and after intervention) (27, 28). Thus, this paper addresses practical and essential questions: How many households are needed in a study group to assess the group's *usual* (“true”) food expenditure pattern or to rank households with reasonable precision? How many weeks of data are needed to precisely evaluate a household's usual food expenditure?

Evaluating a household's usual expenditures requires an understanding of the sources of variation in week-to-week spending. Similar to dietary intake—which varies daily and requires multiple days of assessment—household food expenditures likely vary from week to week, necessitating multiple weeks and adequate sample sizes to ascertain usual food expenditures (28, 29). Group sizes and data collection periods may also vary by food group, analogous to the differing number of dietary assessments needed to evaluate intake of specific nutrients (28).

We quantify within- and between-household variation in weekly expenditures for all foods and beverages and for two specific categories of food: fruits and vegetables, and foods high in added sugar (sugar-sweetened beverages [SSBs], candy, and sweet baked goods). Using these values, we estimate the group size needed to estimate a group's average food expenditures. We also estimate the number of weeks of data needed to rank household expenditures or estimate a household's usual food expenditures with adequate precision. Results from this paper can help researchers design efficient studies of food purchasing behavior (27–29). To our knowledge, this is the first study to provide these important metrics.

## Materials and Methods

### Study Population

This paper is a secondary analysis of data from a prospective trial (26, 30, 31). Briefly, low-income households in the Minneapolis-St. Paul, Minnesota, metropolitan area were recruited between August 2013 and May 2015. Eligibility criteria included: (1) not currently enrolled in the Supplemental Nutrition Assistance Program (SNAP) or planning to enroll during the study; (2) household income <200% the federal poverty rate or participating in a government program that automatically qualifies households for SNAP (e.g., the Diversionary Work Program in Minnesota); (3) adult in the household primarily responsible for food shopping is able to read and speak English and participate in the study. Some SNAP eligibility criteria, such as citizenship status, were not applied. The University of Minnesota Institutional Review Board approved all aspects of the study (ClinicalTrials.gov: NCT02643576).

Participants were asked to annotate and submit all household food purchase receipts throughout the study using the protocol described in greater detail below. At the baseline visit, participants completed a survey to assess demographic characteristics. Household food security was evaluated using the US Household Food Security Survey Module: 6 Item Short Form (32).

Participants who completed baseline measures and submitted at least 2 weeks of receipts received a study debit card with monthly benefits for 12 weeks. Households were randomized into one of four study arms, which varied with respect to whether a financial incentive was provided for fruit and vegetable purchases and whether foods high in added sugars could be purchased with benefits. Analyses for this paper are limited to the baseline period of the trial.

### Simple Annotated Receipt Method

#### Receipt Collection

Research staff met participants in-person to provide verbal and written instructions, and materials necessary for receipt collection. Participants were instructed to collect all food purchase receipts and to query other household members for receipts. Receipts were requested from both restaurants (retailers that serve or sell ready-to-consume food) and food retailers (retailers that primarily sell unprepared food). This paper focuses on receipts from food retailers.

Participants were instructed to annotate food retailer receipts if the item description was vague or unclear. To annotate receipts, participants were instructed to write details directly on the receipt next to the item lacking information. For example, an item described as “produce” would need annotation to specify the type of produce (e.g., “tomatoes”). Annotation was not requested for quantities of food purchased. Missing food receipt forms were requested for purchases without receipts, such as purchases made at retailers that do not provide receipts (e.g., farmer's market) or lost receipts. The missing receipt form included details such as the store name, date of purchase, food items purchased, quantity purchased, price per item, and total price. As part of the instruction process, study staff reviewed a sample annotated receipt and missing receipt form with participants.

All receipts were to be mailed to study staff on a weekly basis using pre-addressed, postage-paid envelopes, which were pre-labeled with the participant ID number, dates comprising the week of receipt collection, and the target mailing date to facilitate tracking by staff. Participants were mailed a gift card as a reward for receipt collection every month. The reward amount was pro-rated, with $30 provided if 4 weeks of receipts were submitted, and lesser amounts for three ($15), two ($10), and one ($5) week. Research staff contacted participants to encourage submission if receipts were not received.

#### Food Retailer Receipt Coding

Receipts were first sorted into two categories: restaurant purchases and food retailer purchases. Restaurants were classified as full-service, limited-service, or unable to determine restaurant type. This study focuses on food retailers, which were further classified as supermarket/market (e.g., Cub, Aldi, farmers market), natural food store (e.g., co-ops), warehouse store (e.g., Costco, Sam's Club), drug store (Walgreens, CVS), convenience store/gas station (including dollar stores), superstore (e.g., Target, Walmart), or other (e.g., Home Depot, Menards) (24). Each receipt was then assigned a unique identifier to specify the participant, week, and receipt number.

Items on food retailer receipts were classified into one of 11 food categories. The choice of food categories reflects the primary aims of the original trial, which was to evaluate two food categories: fruits and vegetables, and foods high in added sugars (sugar-sweetened beverages [SSBs], sweet baked goods, and candies). Items with potential substitution effects (e.g., milk, savory snacks) were measured, while items of lesser interest to the trial (e.g., diet sodas) were categorized as “other food” purchases.

Food items that lacked sufficient detail to code into one of the 11 food categories were coded as having “insufficient detail to code” (unidentified). Before coding an item as unidentified, study staff followed a series of procedures to obtain missing information. First, an online search was conducted using the store name, item, code, and/or abbreviation. When available, the item's Universal Product Codes was searched (http://www.upcdatabase.org). Stores were contacted to verify the item for successful online searches. If these procedures failed to provide necessary details, items were coded as unidentified.

For each receipt, the total number of line items and expenditures were calculated for overall food and beverages, and for each of the food categories. Totals for each category were determined by summing across line items classified into the category. Quantities or weight of foods purchased was not considered in the tabulation. For example, a line item of “apples” would count as a frequency of one in the tally for receipt items for fruits, and total expenditure amounts are reported rather than per unit prices. The first 10 receipts coded were reviewed for accuracy by a second staff member. Errors identified were reviewed and corrected. Spot checks of coded receipts were conducted throughout for quality assurance.

### Statistical Analysis

Analyses for this paper are restricted to participants who submitted at least 3 weeks of food retailer receipts during the 4-week baseline period. First, we described the food purchasing information captured by the method using total number of receipt line items and expenditures for overall food expenditures, each of the 11 food categories, and items categorized as having insufficient detail to code (unidentified). Food purchasing data was also evaluated by store, which was collapsed into four types based on previous literature and low frequency of receipts in some categories: Grocery stores, Convenience stores/Gas Stations, Drug stores, and Superstores/Mass merchandiser/Warehouse club store.

Second, we used a zero-inflated negative binomial model to evaluate whether unidentified line items on receipts were associated with participant characteristic or store type. This model was used because the distribution of unidentifed items was heavily skewed, with 94% of receipts submitted without any unidentified items. Likelihood ratio tests confirmed zero-inflation and overdispersion, supporting the model choice. The model simultaneously evaluates two processes. The logit portion of the model evaluates participant characteristics and store types associated with submitting receipts with unidentified items, yielding odds ratios (OR). The negative binomial model evaluates the incident rate ratios (IRR) of unidentified items by participant characteristics and store types among those who submitted at least one receipt with an unidentified item. Participant characteristics of interest were age, gender, race/ethnicity, marital status, household size, education level, annual household income, and food security. A dummy variable was used to indicate the store type. The model was adjusted for the total number of line items per receipt. A *p* <0.05 was the criterion for claiming statistical significance.

Third, a mixed effects regression model with an unstructured covariance and restricted maximum likelihood estimator was used to estimate the mean, within-household variance ($\sigma_{w}^{2}$), and between-household ($\sigma_{b}^{2}$) variance for overall food expenditures, fruits and vegetables, and foods high in added sugar. We calculated within- and between-household coefficients of variation (CV_w_ and CV_b_, respectively) as percentages using the following equations (29): *CV*_*w*_ = (σ_*w*_/*mean*)*x* 100; *CV*_*b*_ = (σ_*b*_/*mean*)*x* 100. The ratio of within- to between-household variation, the variance ratios, were calculated as $\sigma_{w}^{2}$ /$\sigma_{b}^{2}$ (which is equivalent to ${\left[CV_{w}/CV_{b}\right]}^{2}$).

Using these values, we calculated the group size or weeks of data needed to estimate usual (or “true”) food expenditures with adequate precision. The usual household food expenditures refers to the hypothetical “true” average of the study sample about which a household's expenditures vary during the period of data collection. We assume that within- and between-household variation observed in our sample is due to random variation about the hypothetical average, and not due to a changes in habitual spending patterns (33).

The number of households in a group (n_g_) required to estimate group mean expenditure using a single week of expenditure data was calculated as follows: $n_{g}\;=Z_{\alpha }^{2}x\;\left[\left(CV_{b}^{2}\;+CV_{w}^{2}\right)/D_{0}^{2}\right]$, where D_0_ is a specified percentage deviation of the group's usual expenditure, and Z_α_ is the normal deviate for the percentage of times the measured expenditure should be within a specified limit (29). For the purposes of this study, we evaluated estimates with 95% CIs (i.e., Z_α_ = 1.96), with D_0_ varying between 10 and 50%. The number of weeks of expenditure data (n_r_) needed to obtain a given Pearson correlation coefficient, *r*, between observed and unobserved usual expenditures was also calculated (27, 33). The equation is as follows: $n_{r}\;=\;r^{2}/\left(1-r^{2}\right)x\;\left(\sigma_{w}^{2}\;/\sigma_{b}^{2}\right)$, where *r* varied between 0.75 and 0.95. Finally, the number of weeks of expenditure data (n_w_) required to estimate mean household expenditures within the specified percentage deviation (D_0_) from the household's usual (“true”) expenditure was calculated as follows (29): $n_{w}\;=\;{\left(Z_{\alpha }xCV_{w}/D_{0}\right)}^{2}$. D_0_ varied between 10 and 50% and Z_α_ was fixed at 1.96 to derive 95% CIs.

## Results

Of the 279 participants enrolled in the study, 260 submitted at least 3 weeks of food receipts during the baseline period and were included in the analyses. Participant characteristics are presented in Table 2. To summarize, most participants were female, over half were African-American, and most reported low or very low food security.

**Table 2:** Baseline characteristics of households using the simple annotated receipt method as part of a trial evaluating food purchasing behavior (*n* = 260).

**Characteristic**	***N* (%)**
**AGE, YEARS**	
Under 25	14 (5.4)
25–44	115 (44.2)
45–64	113 (43.5)
Over 65	18 (6.9)
**GENDER**	
Male	48 (18.5)
Female	212 (81.5)
**RACE/ETHNICITY**	
White, Non-Hispanic	77 (29.6)
Black, Non-Hispanic	131 (50.4)
Other, Hispanic	52 (20.0)
**MARITAL STATUS**	
Single, never married	117 (45.2)
Married or partnered	72 (27.8)
Separated/divorced/widowed	70 (27.0)
**HOUSEHOLD SIZE**	
1 person	58 (22.3)
2 people	58 (22.3)
3 people	61 (23.5)
4 or more people	83 (31.9)
**EDUCATION LEVEL**	
High school graduate or less	75 (28.9)
Some college/associates degree	138 (53.1)
College graduate or higher	47 (18.1)
**ANNUAL HOUSEHOLD INCOME**	
$14,999 or less	79 (33.2)
$15,000—$34,999	114 (47.9)
$35,000 or more	45 (18.9)
**HOUSEHOLD FOOD SECURITY STATUS**	
High or marginal	53 (19.3)
Low	96 (34.9)
Very low	126 (45.8)

### Food Purchase Information

Over a 4-week period, households included in the analyses submitted a total of 5,635 receipts. Of these, 2,094 receipts from restaurants and 11 receipts for non-food purchases were excluded from analyses. Over 98% of the receipts were submitted as original receipts; 1.5% (*n* = 52) were submitted as missing receipt forms. Over the 4-week data collection period, households submitted on average 13.6 receipts (95% CI: 12.5, 14.7). This translates to 3.4 receipts per week (95% CI: 3.1, 3.7), with an average of 8.3 (95% CI: 7.6, 9.0) line items per receipt. On average, households spent $23.30 (95% CI: $21.00, $25.51) per receipt.

**Figure 1:**
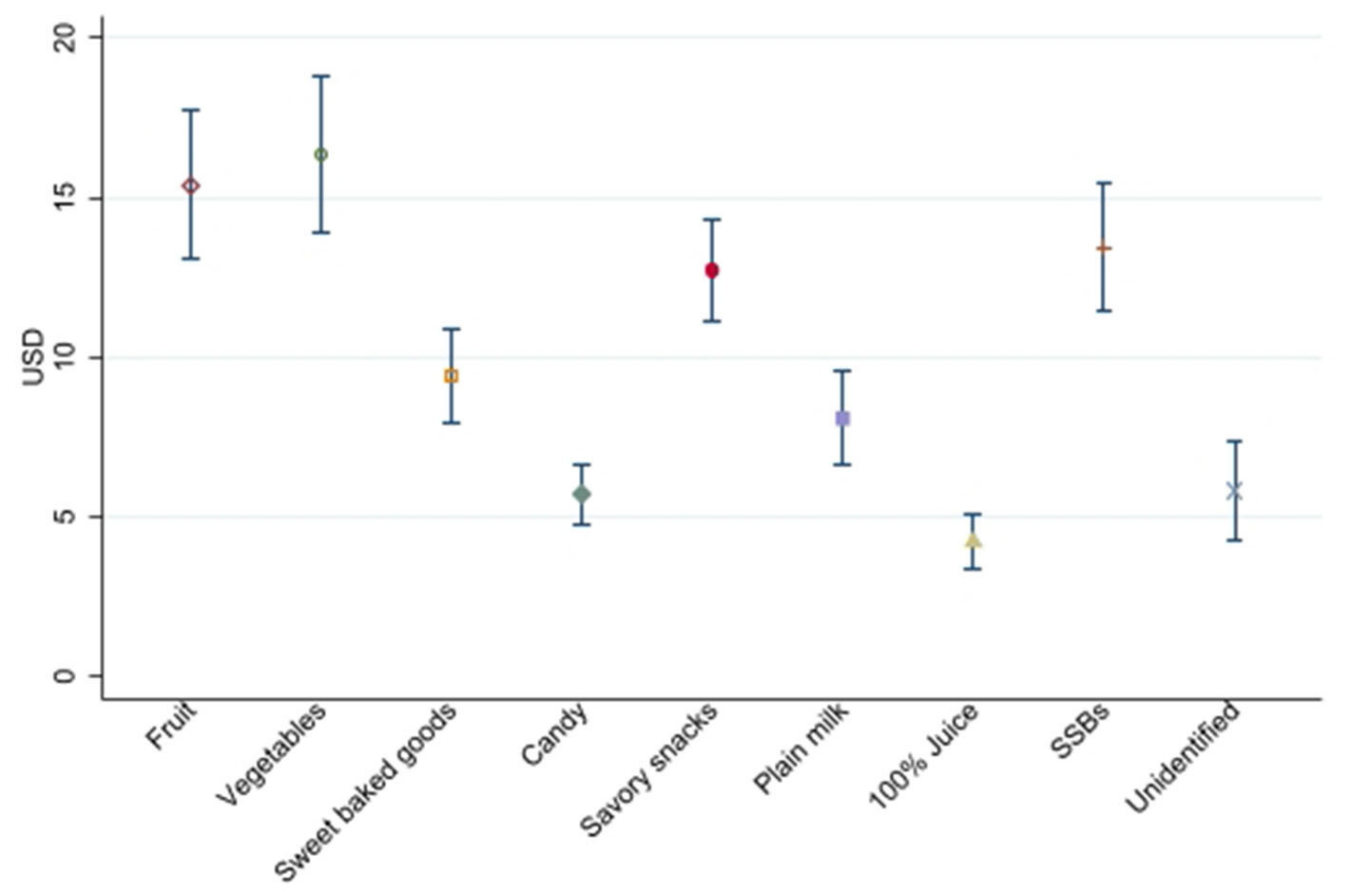
Average household expenditures submitted over a 4-week period by low-income households using a simple annotated receipt method, for selected food categories (*n* = 260).

Figure 1 shows the average household food expenditures over a 4-week period for selected food categories. On average, unidentified items accounted for $5.81 (95% CI: $4.25, $7.37) of household food expenditures over the 4-week period. Fruit and vegetable expenditures accounted for $15.41 (95% CI: $13.04, $17.77) and $16.34 ($13.90, $18.78), respectively. Households spent an average of $13.45 (95% CI: $11.45, $15.45) on sugar-sweetened beverages (SSBs) over the 4-week period. Table 3 presents average household food expenditures and line items submitted over a 4-week period for all categories of food. Foods coded as “other” –food and beverages that did not fit into the 11 food categories of interest—comprised the largest share of expenditures, accounting for $180.00 (95% CI: $162.52, $197.47) of food expenditures and 58.7 (95% CI: 53.2, 64.2) receipt line items over a 4-week period.

**Table 3:** Average household food expenditures and receipt line items submitted over a 4-week period by low-income households using a simple annotated receipt method, by food category and store type (*n* = 260).

	**Expenditures USD (95% CI)**	**Receipt line items number (95% CI)**
*Total*	*272.62 (247.13, 298.11)*	*98.8 (90.1, 107.6)*
**FOOD CATEGORY**		
Fruits	15.41 (13.05, 17.77)	5.6 (4.9, 6.3)
Vegetables	16.34 (13.90, 17.78)	9.0 (7.7, 10.2)
Sweet baked goods	9.39 (7.94, 10.85)	3.6 (3.1, 4.1)
Candy	5.70 (4.75, 6.65)	3.36 (2.8, 3. 9)
Savory snacks	12.72 (11.15, 14.29)	5.4 (4.8, 6.0)
Regular, unflavored milk	8.07 (6.59, 9.55)	2.5 (2.1, 2.9)
Flavored milk	0.47 (0.23, 0.71)	0.2 (0.1, 0.3)
100% Juice	4.21 (3.33, 5.10)	1.5 (1.2, 1.8)
Sugar-sweetened beverages	13.45 (11.44, 15.45)	7.1 (6.1, 8.2)
Fruit beverage, unknown type	1.04 (0.62, 1.46)	0.4 (0.3, 0.5)
Other foods	180.00 (162.52, 197.47)	58.7 (53.2, 64.2)
Unidentified	5.81 (4.25, 7.37)	1.6 (1.2, 2.0)
**STORE TYPE**		
Grocery stores	169.34 (150.37, 188.30)	62.4 (55.5, 69.3)
Superstores/Mass merchandisers	115.68 (98.89, 132.46)	36.9 (31.7, 42.0)
Convenience stores/Gas stations	18.30 (15.02, 24.57)	11.7 (9.7, 11.6)
Drug stores	10.38 (7.82, 12.94)	5.0 (3.9, 6.1)

**Figure 2:**
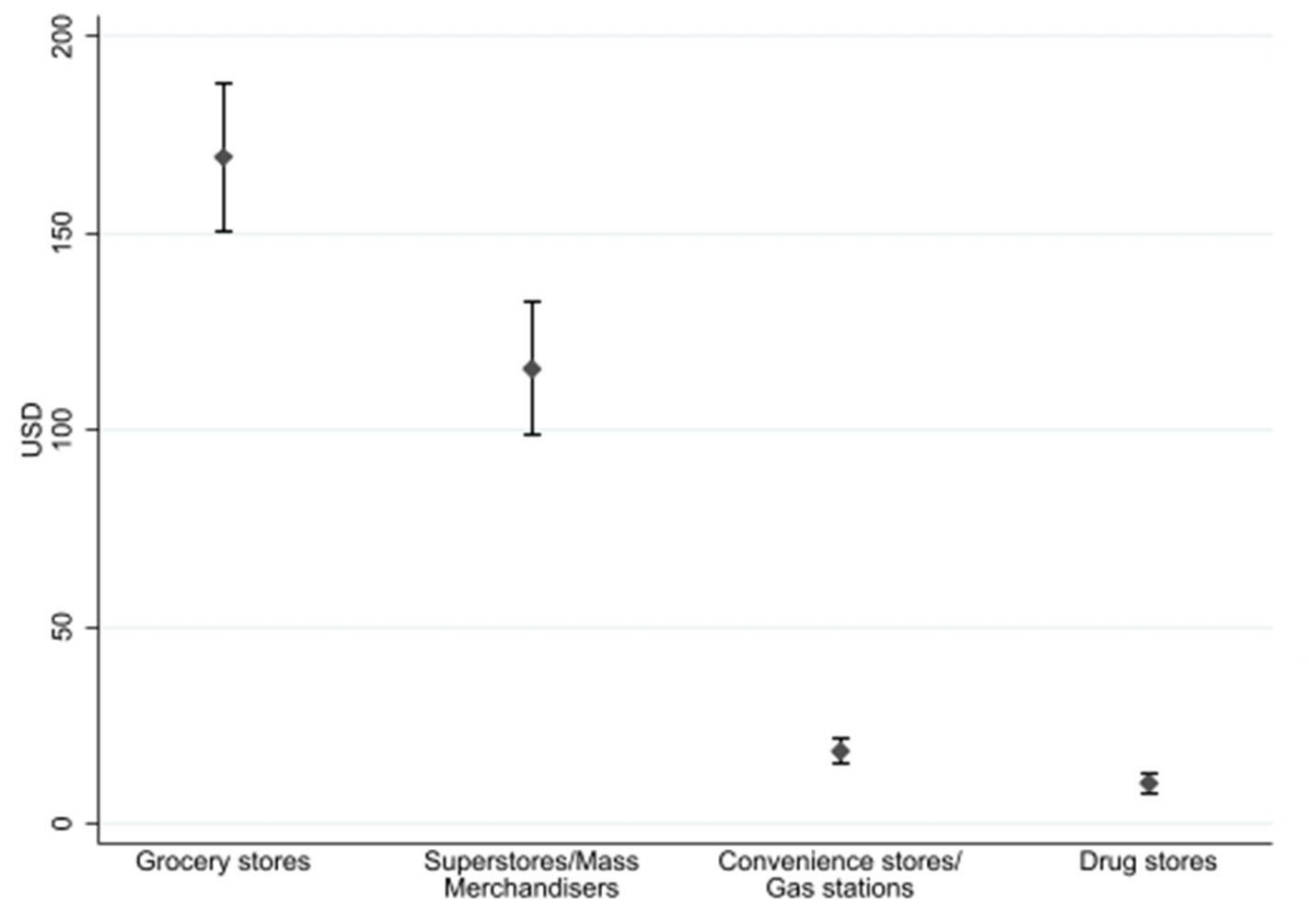
Average household expenditures submitted over a 4-week period by low-income households using a simple annotated receipt method, by store type (*n* = 260).

Figure 2 shows the average household food expenditures over a four-week period by store type. Households spent the most money at grocery stores ($169.34, 95% CI: $150.37, $188.30) and superstores/mass merchandisers/warehouse club stores ($115.68, 95% CI: $98.89, $132.46). Table 3 presents average household food expenditures and receipt line items submitted over a 4-week period by different store types. Grocery stores and superstores accounted for the greatest number of line items submitted over the 4-week period, accounting for 62.4 (95% CI: 55.5, 69.3) and 36.9 (95% CI: 31.7, 42.1) receipt line items, respectively.

Supplemental Tables 1, 2 describe the total volume of food expenditure information captured over the 4-week data collection period. The 3,530 food retailer receipts submitted by the study sample represented $70,822.21 in total food expenditures and contained over 25,000 line items. Food purchases coded as “other” —food and beverages that do not fit into the 11 coded food categories of interest—comprised the largest share of expenditures at food retailers (66.0%), followed by vegetables (6.0%), fruits (5.7%), sugar sweetened beverages (4.9%), and savory snacks (4.7%). With respect to findings for the number of receipt line items, “other” composed the largest number of line items (59.4%) followed by vegetables (9.1%), sugar sweetened beverages (7.2%), fruits (5.7%), and savory snacks (5.5%). “Unsure fruit beverages” (fruit beverages for which it could not be determined whether the beverage was 100% fruit juice or a fruit drink that should be classified as a sugar sweetened beverage) comprised <1 percent of both the total food spending (0.4%) and the proportion of total line items (0.4%) (Supplemental Table 1). Nearly 60% of total food retailer expenditures ($41,826.57) was spent in supermarkets/markets, and $24,985.98 (35.8%) was spent in superstores/mass merchandisers/warehouse club stores (Supplemental Table 2).

### Unidentified Food Expenditures

Unidentified food expenditures comprised 2.1% of total spending and 1.6% of total line items submitted by 260 households over a 4-week period (Supplemental Table 1). Table 4 presents results from the zero-inflated negative binomial model to evaluate the association between unidentified receipt items and participant characteristic and store type. Drugs stores had a lower rate of unidentified line items compared to supermarkets (*p* = 0.04). There were no significant differences in the rate of occurrence of unidentified receipt line items by the participant characteristics examined.

**Table 4:** Adjusted^*^ incidence rate ratios of unidentified items in receipts submitted over a 4-week baseline period by 260 low-income households using a simple annotated receipt method (*n* = 3,530 food retailer receipts).

	**Unidentified receipt line items IRR (95% CI)**
**STORE TYPE**	
Supermarket (ref)	1.00
Convenience store/Gas station	1.03 (0.58, 1.82)
Drug store	0.11 (0.01, 0.99)
Superstore/Mass merchandiser/Warehouse club store	0.92 (0.47, 1.80)
**AGE, YEARS**	
<25	1.40 (0.67, 2.91)
25–44 (ref)	1.00
45–64	1.56 (0.67, 3.66)
Over 65	1.04 (1.90, 5.70)
**GENDER**	
Female (ref)	1.00
Male	1.11 (0.70, 1.82)
**RACE/ETHNICITY**	
White, Non-Hispanic (ref)	1.00
Black, Non-Hispanic	0.65 (0.37, 1.13)
Other, Hispanic	1.40 (0.71, 2.62)
**MARITAL STATUS**	
Single, never married (ref)	1.00
Married or partnered	0.76 (0.37, 1.13)
Separated/divorced/widowed	1.37 (0.71, 2.62)
**HOUSEHOLD SIZE**	
1 person (ref)	0.40 (0.19, 0.84)
2 people	0.68 (0.30, 1.60)
3 people	0.68 (0.41, 1.12)
4 or more	1.00 (ref)
**EDUCATION LEVEL**	
High school graduate or less	1.00
Some college/associates degree (ref)	0.78 (0.50, 1.23)
College graduate or higher	1.14 (0.58, 2.23)
**ANNUAL HOUSEHOLD INCOME**	
$14,999 or less	1.00
$15,000–$34,999 (ref)	0.79 (0.50, 1.26)
$35,000 or more	0.87 (0.46, 1.64)
**FOOD SECURITY**	
Very low (ref)	1.00
Low	0.67 (0.44, 1.04)
High or marginal	0.65 (0.33, 1.27)

* *Model adjusted for the total number of line items per receipt*.

### Within- and Between-Household Variation

Table 5 shows the means, within-household coefficient of variation (CV_w_), between-household coefficient of variation (CV_b_), and ratios for weekly household expenditures for total food expenditures and selected food categories. On average, households spent $85.65 per week (standard error of the mean [SE] $5.38) on total food expenditures. Mean household expenditures on fruits and vegetables was $11.05 per week (SE $0.90), with comparable amounts spent on fruits and vegetables individually. Households spent an average of $7.95 (SE $0.78) per week on foods high in added sugar, with varying amounts spent on individual food categories. Regardless of food category, CV_w_ was larger than CV_b_, and both values were higher when evaluating individual food categories for foods high in added sugar. The CV ratio was above 1 for all categories of food, ranging from 1.44 for fruits and vegetables to 6.44 for candy.

**Table 5:** Mean weekly expenditures (USD), within-household coefficients of variation (CV_w_), between-household coefficients of variation (CV_b_), and variance ratios for food expenditures of low-income households using the simple annotated receipt method (*n* = 260).

	**Mean (SE) Dollars (USD)/week**	**CV_**w**_**	**CV_**b**_**	**Variance Ratio**
*Total food expenditures*	85.65 (5.38)	70.8	49.8	2.02
*Fruits and vegetables*	11.05 (0.90)	85.5	71.3	1.44
Fruits	5.67 (0.53)	100.5	75.6	1.77
Vegetables	5.38 (0.52)	104.4	81.3	1.65
*Foods high in added sugar*	7.95 (0.78)	105.6	79.0	1.78
Sugar-sweetened beverages	3.57 (0.45)	138.3	95.5	2.10
Sweet baked goods	2.70 (0.37)	161.9	80.3	4.07
Candy	1.70 (0.26)	184.5	72.7	6.44

*CV_w_, Within-household coefficient of variation; CV_b_, Between-household coefficient of variation; Variance ratio, $\sigma_{w}^{2}$ /$\sigma_{b}^{2}$ = (CV_w_/CV_b_)^2^*.

Table 6 shows the number of households in a group required to estimate the group mean weekly expenditure with 95% CIs within 10–50% deviation of the group's observed mean from the group's usual (“true”) mean. To maintain precision of ±20% of the group's true total food expenditures, at least 72 households are required. Larger group sizes are required to estimate specific food categories, with the highest requirements for evaluating individual categories of food high in added sugar.

**Table 6:** Number of households in a group needed to estimate weekly expenditures with 95% CIs within 10–50% deviation of the observed group mean from the group's usual (“true”) mean using a single week of expenditure data.

	**Specified % of true mean**				
	**10%**	**20%**	**30%**	**40%**	**50%**
*Total food expenditures*	288	72	32	18	12
*Fruits and vegetables*	477	119	53	30	19
Fruits	609	152	68	38	24
Vegetables	674	168	75	42	27
*Foods high in added sugar*	669	167	74	42	27
Sugar-sweetened beverages	1087	272	121	68	43
Sweet baked goods	1256	314	140	79	50
Candy	1514	379	168	95	61

Table 7 shows the number of weeks of food expenditure data required to ensure a correlation coefficient, *r*, between observed and true expenditures. As the variance ratio decreased, fewer weeks of observation were needed to rank households by expenditure and distinguish households with low expenditures from those with high expenditures. Assuming *r* = 0.90, a minimum of 9 weeks of data are required for total food expenditures, 6 weeks of data for fruits and vegetables, and 8 weeks of data for foods high in added sugar. Compared to evaluating fruits and vegetables as individual food categories, a greater number of weeks are required for evaluating individual categories of food high in added sugar to rank households with a given *r*.

**Table 7:** Number of weeks of data needed to ensure a given correlation coefficient, *r*, between observed and usual (“true”) weekly household expenditures.

	***r*****-value**				
	**0.75**	**0.80**	**0.85**	**0.90**	**0.95**
*Total food expenditures*	3	4	5	9	19
*Fruits and vegetables*	2	3	4	6	13
Fruits	2	3	5	8	16
Vegetables	2	3	4	7	15
*Foods high in added sugar*	2	3	5	8	17
Sugar-sweetened beverages	3	4	5	9	19
Sweet baked goods	5	7	11	17	38
Candy	8	11	17	27	60

Table 8 shows the number of weeks of food expenditure data required to estimate mean weekly household expenditures with 95% CIs within 10–50% deviation from the usual (“true”) household expenditure. To maintain precision of 20% within the household's true expenditures, 48 weeks are required to estimate total food expenditures with 95% CIs. Greater number of replicate weeks of data are required to estimate individual food categories, with the highest number of weeks for categories of foods high in added sugar.

**Table 8:** Number of weeks of data needed to estimate mean household expenditures with 95% CIs within 10–50% of the usual (“true”) household mean.

	**Specified % of usual (true) mean**				
	**10%**	**20%**	**30%**	**40%**	**50%**
*Total food expenditures*	192	48	21	12	8
*Fruits and vegetables*	281	70	31	18	11
Fruits	388	97	43	24	16
Vegetables	419	105	47	26	17
*Foods high in added sugar*	428	107	48	27	17
Sugar-sweetened beverages	734	184	82	46	29
Sweet baked goods	1006	252	112	63	40
Candy	1308	327	145	82	52

## Discussion

This paper describes and evaluates a simple annotated receipt method for assessing household food purchasing. Results show that the method can capture food purchasing information for various food categories in a variety of store types, and may be a feasible tool for use among diverse, low-income populations.

Most food items on the receipts could be coded into one of the 11 food categories of interest in the study. Only 1.6% of line items—comprising 2.1% of total spending—could not be categorized because of insufficient detail. Importantly, unidentified line items did not vary by demographic characteristics, which suggests that the tool is applicable to diverse, low-income populations. Compared to supermarkets, drug stores had a lower rate of unidentified items. This may be because drugs stores tend to sell less produce, fresh meats, and bulk items, which often lack detail on receipts and require less annotation by the participant.

Findings also suggest that the simple annotated receipt method may be adapted for specific research questions. While the majority of food items were coded as “other,” this is a result of a priori food category definitions outlined in the study protocol. The experimental trial for which this method was developed assessed policy changes to SNAP. As a result, the focus was on policy-specific food categories—specifically, fruits, vegetables, sweet baked goods, sugary sweetened beverages, and candies. The “other” category captured foods that were of lesser interest to the study aims, such as diet sodas and water. However, this category is adaptable to various study-specific questions. For example, sugar-sweetened beverages (SSBs) and fruit juices were of interest in the study. To ensure comprehensive and precise evaluation of beverage expenditures, multiple categories of beverages were specified, including a “fruit beverage, unknown type” category for fruit beverages that could not be identified as either 100% fruit juice or a sugar-sweetened fruit drink. Items labeled “fruit beverage, unknown type” comprised only 0.4% of receipt line items in comparison to 7.2% of line items for SSB and 1.5% of line items for fruit juices, suggesting that the present method can differentiate food and beverage categories as required by study-specific aims. Researchers interested in capturing different food or beverage categories can therefore adapt the method to study-specific needs using different coding protocols (e.g., “diet sodas” were included in the “other” category in the present study, but can be coded).

To our knowledge, this is the first study to apply established methods of evaluating within- and between-individual variations to a food expenditure assessment tool (27–29). The results have implications for the design of studies evaluating household food expenditures in lower-income households. CV_w_ and CV_b_ values were lowest for total food expenditures and largest for individual categories of foods high in added sugar. Larger CV_w_ values for foods high in added sugar values had the greatest impact on the number of replicate weeks required to assess a household's usual food expenditures. For example, candy had the highest CV_w_ value of the food categories evaluated, requiring 52 to 1,307 weeks to estimate the household mean weekly expenditure within 10–50% deviation of the true values. Future researchers should consider alternative or additional tools to evaluate expenditures of foods such as SSBs, candies, and sweet baked goods that are highly variably purchased week to week by households.

Our findings also suggest that the simplified annotated food receipt method is most appropriate for comparing mean expenditures of different study groups or ranking household expenditures (e.g., into quartiles). For example, a group size of at least 119 households is required to estimate the mean group expenditure on fruits and vegetables within 20% of the true mean. Similarly, at least 6 weeks of data are required to rank households by weekly fruit and vegetable expenditure level with a precision of *r* = 0.90.

### Strengths and Limitations

Food purchasing behavior is strongly patterned by socioeconomic status (7, 8), but few food receipt methods have been evaluated in low-income households (12). This study addresses the need for feasible methods to evaluate food purchasing. Importantly, this novel method was evaluated in a sample of diverse, low-income households. This study also has a relatively large sample size and prolonged duration of receipt collection for evaluation of a measurement method.

There are several limitations worth noting. This study did not assess the completeness of receipt submission, the accuracy of receipt annotation, or the reliability of coding. It is possible that receipts were not submitted for some food purchases, resulting incomplete assessment of food purchasing. Future evaluations of this methodology should evaluate completeness of receipt submission and evaluate inter-rater reliability of receipt coding. Furthermore, this receipt method does not provide information on food quantities. Expenditure data may suffice if change in food purchasing is the primary outcome of interest (e.g., to evaluate whether an intervention decreases purchasing of SSBs). Previous studies also suggest that food expenditure data may be a reasonable approximation of intake (17, 19, 21). Evaluating the association of expenditure data with food quantities and dietary intake is an area for further method development. The present analyses also relies on a sample of lower-income households in one metropolitan area. The levels of variation in food expenditures may differ for other population groups and requires further research.

Finally, the present method was not directly compared to other receipt methods. A qualitative review of previously published studies shows that results are somewhat comparable. This suggests that the present method may be able to capture details similar to previous receipt methods—while potentially reducing the burden for participants (compared to the annotated receipt method) and minimizing the number of unidentified food expenditures (compared to the receipt collection method). A study using the annotated receipt collection method, which requires transcription of all receipt information, collected an average of 3.1 receipts from food retailers per household per week (24). This is comparable to an average of 3.3 receipts per household per week in the present study. The annotated receipt method also yielded an average of 25.8 line items per household per week for both food retailers and restaurant receipts (24)—compared to 24.7 line items per household per week in the present study, which included only food retailers. Results for specific beverage categories across receipt methods also suggests similarities. Sugar-sweetened beverages accounted for 9.1% of all line items using the annotated receipt method, compared to 7.2% in the present study (24). In a study using the receipt collection method—which involves neither annotation or transcription-−100% fruit juices comprised 1.6% of total grocery expenditures, similar to 1.6% of total expenditures in the present study (22). Importantly, the present method may have a lower rate of “missing/unclassified” items compared to the receipt collection method, which was previously reported as having 7.7% “missing classified/unclassified” expenditures (22).

However, it is worth noting that the annotated receipt method and receipt collection methods discussed above were deployed in different populations and studies. The annotated receipt method followed 90 participants who were predominantly white women in Minneapolis, Minnesota, for 4 weeks. In contrast, the receipt collection method was used for a sample of 107 diverse, low-income households in Houston, Texas over a 6-weeks period. The present study is specific to ethnically and racially diverse households in the Minneapolis-St. Paul, Minnesota, metropolitan area. Future studies are needed to formally compare different methods.

## Conclusions

The simple annotated food purchase receipt method is a promising approach for assessing food purchasing behavior. Our findings suggest that this method is able to capture a wide range of food purchasing information from a variety of store types. Unidentified items were limited and did not vary by participant characteristic or stores, suggesting that the present method is broadly applicable among diverse, low-income households. This paper is also the first to quantify within- and between-household variation in food expenditures using a receipt method, which is crucial information for determining sample sizes, estimating data collection periods, and interpreting findings. Research is needed to further evaluate the method and compare it to alternative receipt methods to assess food purchasing behavior.

## Data Availability Statement

The raw data supporting the conclusions of this article will be made available by the authors, without undue reservation.

## Ethics Statement

The studies involving human participants were reviewed and approved by The University of Minnesota Institutional Review Board. The patients/participants provided their written informed consent to participate in this study.

## Author Contributions

SV performed statistical analyses. SV and LH wrote the first draft of the manuscript. All authors read and approved the final manuscript. All authors contributed to the article and approved the submitted version.

## Conflict of Interest

The authors declare that the research was conducted in the absence of any commercial or financial relationships that could be construed as a potential conflict of interest.
