# Supplementary material for: Within- and Between-Household Variation in Food Expenditures Among Low-Income Households Using a Novel Simple Annotated Receipt Method
Source: Front Nutr. 2020 Oct 22;7:582999. doi: 10.3389/fnut.2020.582999 (PMC7642585; doi:10.3389/fnut.2020.582999)
Supplement: Supplementary file 1 [file Table_1.DOCX]

# **Supplemental Table 1**. Total number of receipt line items and expenditures submitted over a four-week baseline period by 260 low-income households using a simple annotated receipt method (n=3,530 food retailer receipts)

| **Food category** | **Receipt line items**  **Number (% of total)** | **Expenditures  US$ (% of total)** |
| --- | --- | --- |
| Fruits | 1457 (5.7) | 4006.80 (5.7) |
| Vegetables | 2331 (9.1) | 4248.96 (6.0) |
| Sweet baked goods | 933 (3.6) | 2442.35 (3.4) |
| Candy | 874 (3.4) | 1481.50 (2.1) |
| Savory snacks | 1406 (5.5) | 3307.74 (4.7) |
| Regular, unflavored milk | 639 (2.5) | 2099.30 (3.0) |
| Flavored milk | 48 (0.2) | 122.84 (0.2) |
| 100% Juice | 397 (1.5) | 1095.27 (1.5) |
| Sugar-sweetened beverages | 1849 (7.2) | 3497.22 (4.9) |
| Fruit beverage, unknown type | 97 (0.4) | 271.29 (0.4) |
| Other foods | 15255 (59.4) | 46798.92 (66.0) |
| Unidentified | 413 (1.6) | 1510.02 (2.1) |
| *Total* | *25699* | *70882.21* |

# **Supplemental Table 2**. Total number of receipts and food expenditures by store type submitted over a four-week baseline period by 260 low-income households using a simple annotated receipt method (n=3,530 food retailer receipts)

| **Food retailer type** | **Number of receipts  Number (% of total)** | **Expenditures**  **USD (% of total)** |
| --- | --- | --- |
| Supermarket/Market | 1859 (52.8) | 41826.57 (59.0) |
| Convenience store/Gas station | 571 (16.2) | 2854.45(4.0) |
| Drug store | 153 (4.3) | 820.30(1.2) |
| Superstore/Mass merchandisers/Warehouse club store | 940 (26.7) | 24985.98 (35.8) |
